# Effects of astaxanthin supplementation during vitrification and liquid nitrogen vapor freezing on motility, morphology, survival, reactive oxygen species (ROS), and DNA fragmentation of post-cryopreserved human sperm

**DOI:** 10.5935/1518-0557.20240056

**Published:** 2024

**Authors:** Sitthanan Thanintranon, Ubol Saeng-anan, Teraporn Vutyavanich, Waraporn Piromlertamorn, Pareeya Somsak, Usanee Sanmee

**Affiliations:** 1 Division of Reproductive Medicine, Department of Obstetrics and Gynecology, Faculty of Medicine, Chiang Mai University, Chiang Mai 50200, Thailand; 2 CMEx Fertility Center, Center of Medical Excellence, Chiang Mai University, Chiang Mai 50200, Thailand

**Keywords:** astaxanthin, sperm cryopreservation, vitrification, vapor freezing

## Abstract

**Objective:**

To investigate the effect of astaxanthin supplementation in cryopreservation media on post-thawed sperm motility, viability, morphology, reactive oxygen species (ROS), and DNA fragmentation in two cryopreservation techniques using vitrification and liquid nitrogen vapor freezing.

**Methods:**

Thirty normozoospermic semen samples were used in the study. Post-prepared semen samples were divided into 1) non-cryopreserved control, 2) and 3) vitrified without (V) and with astaxanthin 0.5 µM (V+ATX), 4) and 5) frozen in liquid nitrogen vapor without (L) and with astaxanthin 0.5 µM (L+ATX).

**Results:**

Cryopreservation using vitrification and liquid nitrogen vapor freezing significantly decreased sperm motility and viability and increased ROS levels. However, no changes were seen in sperm morphology or DNA fragmentation. The addition of astaxanthin in cryopreservation media significantly increased post-thawed motility in both vitrification (77.6±8.9% vs. 69.0±9.5% in V+ATX and V) and vapor freezing (57.0±13.3% vs. 47.7±14.6% in L+ATX and L); it significantly increased sperm viability in vitrification (75.0±11.9% vs. 65.9±11.1% in V+ATX and V), and significantly decreased ROS level in both vitrification (4.7 (2.6-8.3) RLU/sec/106 vs. 10.6 (9.4-16.0) RLU/sec/10^6^ in V+ATX and V) and vapor freezing (4.6 (3.3-10.5) RLU/sec/106 vs. 10.3 (7.9-18.6) RLU/ sec/10^6^ in L+ATX and L). Astaxanthin supplementation in cryopreservation media did not affect sperm morphology or DNA fragmentation.

**Conclusions:**

Astaxanthin supplementation improved post-cryopreserved sperm motility, decreased ROS levels in both vitrification and liquid nitrogen vapor freezing and improved sperm viability only in the vitrification technique.

## INTRODUCTION

Human sperm cryopreservation is nowadays a standard procedure in assisted reproductive technology (ART) centers worldwide. Two main cryopreservation methods are employed, namely slow vapor freezing and vitrification. The two methods are equally effective. Slow freezing was introduced by Behrman & Sawada (1966). The technique involves gradually decreasing temperatures for two to four hours, manually or automatically, with a programmable freezer, before storing sperm samples in liquid nitrogen at -196°C (Thachil & Jewett, 1981). In 2010, our team developed a vitrification technique as a new alternative method for sperm cryopreservation (Vutyavanich *et al.*, 2010). The medium was modified to be viscous and less toxic. A solid surface vitrification system was used to avoid direct contact of sperm with liquid nitrogen, prevent the boiling effect from occurring, and allow a more uniform cooling rate. A pre-cooled aluminum block was used as a cooling device. The method significantly improved sperm survival and motility and decreased DNA fragmentation compared to conventional slow freezing.

The concept of reactive oxygen species (ROS) has been recently introduced in the ART field. Excessive ROS production induces oxidative DNA and plasma membrane damage, which eventually results in reduced post-thawing sperm motility and viability, subsequently causing poor fertilization and embryo development (Guthrie & Welch, 2012; Gualtieri *et al.*, 2021). Cryopreservation of delicate human gametes produces ROS and, hence, sperm damage (Kim *et al.*, 2011; Ozimic *et al.*, 2023). Antioxidants have been shown to improve the outcomes of sperm cryo-preservation by reducing ROS production and preventing cellular damage. Several agents have been used as supplements, including glutathione, ascorbic acid, vitamin E, L-glutamine, L-cysteine, melatonin, and zinc (Taylor *et al.*, 2009; Bahmyari *et al.*, 2020; Hussein *et al.*, 2023).

This study focused on astaxanthin, a natural substance of the carotenoid group with a structure similar to beta-carotene. It can be found in the seaweed of the *Haematococcus pluvialis* species, as well as other ocean fish (Goto *et al.*, 2001). Astaxanthin is a potent antioxidant that enhances post-thaw sperm quality in humans (Dede & Saylan, 2022; Ghantabpour *et al.*, 2022), boars (Lee & Kim, 2018; Guo *et al.*, 2021), and dogs (Qamar *et al.*, 2020). However, the effect of astaxanthin supplementation in sperm vitrification has not been investigated yet.

Therefore, this study investigated the antioxidative property of astaxanthin in human sperm cryopreservation with vitrification and liquid nitrogen vapor freezing on post-thaw sperm motility, viability, morphology, DNA fragmentation, and ROS production.

## MATERIAL AND METHODS

### Participant and semen collection

Leftover semen samples were collected from male patients aged 20-45 who came to the CMEx Fertility Center at Maharaj Nakorn Chiang Mai Hospital. The patients were handed sterile containers for sperm collection. Semen was collected by masturbation after 2-7 days of abstinence. Only semen with standard parameters, as defined by the World Health Organization (WHO, 2021), were included in the study (sperm volume ≥ 1.4 ml, concentration ≥ 16 x 10^6^ cells/ml, total sperm motility ≥ 42%, progressive motility ≥ 30%, normal morphology ≥ 4%, and leukocyte count < 1.0 x10^6^ cell/ml). Study participants signed an informed consent term before sample collection. This study was approved by the Research Ethics Committee of the School of Medicine, Chiang Mai University, and granted certificate no. 320/2562.

### Experimental design

**Figure 1:**
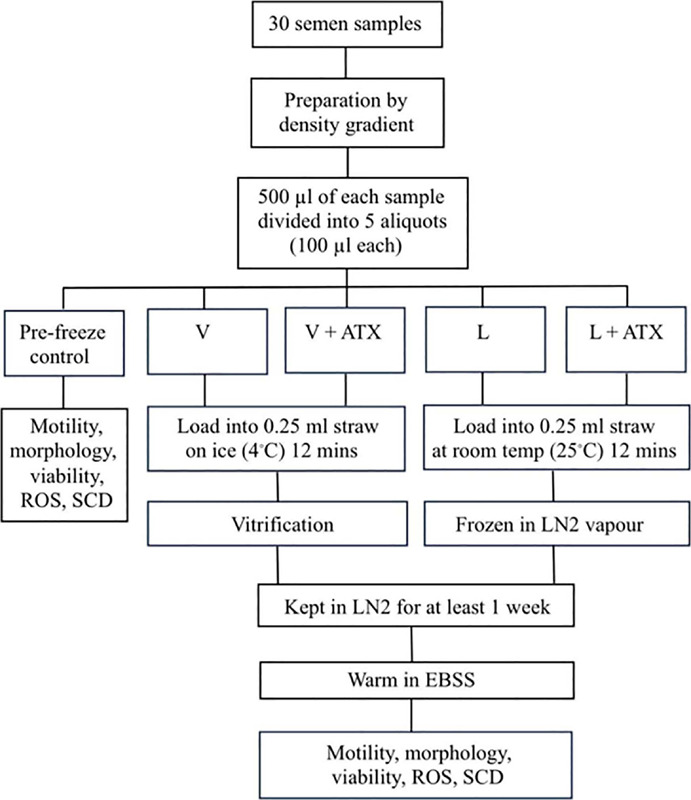
Study flowchart. Abbreviation: V = vitrification, L = liquid nitrogen vapor freezing, ATX = astaxanthin, LN2 = liquid nitrogen, ROS = reactive oxygen species, SCD = sperm chromatin dispersion test.

Samples were prepared using the density gradient centrifugation method. Semen samples were layered on top of 80% and 40% discontinuous Sil-Select Plus gradients (Fertipro N V, Beernem, Belgium), and then centrifuged at 350 g for ten minutes. The sperm pellet was washed twice with 4 ml of Earle’s Balanced Salt Solution (EBSS; Biological Industries, Kibbutz Beit Haemek, Israel), supplemented with 0.3% human serum albumin (HSA; Life Global, Guilford, CT), 0.03M sodium pyruvate (Cat. No. P5280; Sigma Chemical Company, St. Louis, MO) and 1M HEPES (Cat. No. H0887; Sigma) and centrifuged at 200 g for five minutes. The supernatant was discarded, and the final pellet was resuspended in 500 µl of the same medium and divided into five aliquots. The first aliquot (100 µl) served as a non-frozen control and was immediately assessed for sperm motility, kinetics, morphology, viability, ROS levels, and DNA integrity. The remaining four aliquots were cryopreserved by vitrification or liquid nitrogen vapor freezing with or without astaxanthin supplementation in the cryopreservation media. The study flow chart is shown in Figure 1. Astaxanthin (SML0982; Sigma Chemical Company, St. Louis, MO) was dissolved in dimethyl sulfoxide (DMSO; Sigma-Aldrich, St Louis, MO, USA), and a concentration of 0.5 µM was used in this study.

### Vitrification and warming

Vitrification was performed on the second and third al-iquots. The aliquots, containing 100 µl of prepared semen, were mixed dropwise with an equal volume of cryopro-tective medium. The second aliquot was mixed with plain cryoprotectant, while the third was mixed with the same cryoprotectant supplemented with 0.5 µM astaxanthin. The in-house-made medium contained 10% glycerol, 10% HSA, 133 mM glycine, 5.5 mM glucose, 100 mM Trehalose, 12.2 mM sodium pyruvate, and 20 mM HEPES. The mixtures were loaded into 0.25 ml straws, incubated at 4˚C for 12 minutes, and then inserted into a pre-cooled, in-house-developed aluminum block previously submersed in liquid nitrogen (Vutyavanich *et al.*, 2010). For warming, the straws were warmed with water at room temperature (25-28˚C). The samples were then washed with EBSS, and centrifuged at 200 g for five minutes to remove cryo-protective agents. Warmed samples were immediately assessed for sperm motility, kinetics, morphology, viability, ROS level, and DNA integrity.

### Liquid nitrogen vapor freezing and thawing

Liquid nitrogen vapor freezing was performed on the fourth and fifth aliquots. They were mixed dropwise with an equal volume of warm (37˚C) cryoprotective medium (Sperm Freezing, Lifeglobal, USA), without (fourth aliquot) or with 0.5 µM of astaxanthin (fifth aliquot). The mixtures were loaded into straws, incubated at room temperature for 12 minutes, then placed in a horizontal position 10 cm above liquid nitrogen for 12 minutes, according to manufacturers’ instructions. For thawing, the straws were placed in warm (37˚C) water. The samples were then washed in EBSS and immediately assessed for sperm motility, kinetics, morphology, viability, ROS level, and DNA integrity.

### Sperm assessment

Sperm motility and kinetics were assessed using an HTM IVOS II computer-assisted semen analyzer (CASA; Hamilton Thorne Biosciences, Beverly, MA) equipped with the Clinical Human Motility II software. The kinematic parameters measured included the velocity of smooth average cell path (VAP), mean curvilinear velocity (VCL), mean straight-line velocity (VSL), the amplitude of lateral head displacement (ALH), percent linearity (LIN= VSL/VCL x100), and percent straightness (STR=VSL/VAPx100).

For sperm morphology assessment, the washed samples were smeared on glass slides and labeled accordingly. They were stained with Diff-Quick and assessed with an HTM IVOS II computer-assisted semen analyzer (CASA; Hamilton Thorne Biosciences, Beverly, MA). At least 200 spermatozoa were read in duplicate on every slide.

For sperm viability assessment, 10 µl from each aliquot was mixed with 10 µl of 0.5% Eosin-Y (Sigma Chemical) on a glass microscopic slide. Viable sperm appear unstained, whereas the stained ones (red) are dead. At least 200 spermatozoa were counted in duplicate.

The ROS level was assessed by a chemiluminescence technique using a Glomax 20/20 luminometer (Turner Biosystems Inc., Sunnyvale, CA, USA). In essence, ROS and specific reagents react and emit photons that pass through the photo-multiplier tubes of the luminometer. The results are measured as relative light units (RLU) of counted photons per minute (CPM) or mV/s. The reagent was prepared using 20 µl of luminol stock solution (5-amino-2,3 dihydro-1,4 phthalazinedione, Cat. No A8511; Sigma Chemical) mixed with 380 µl of DMSO (Cat. No. D8779; Sigma Chemical) in a foil-covered polystyrene tube. The positive control was a mixture of 400 µl of phosphatebuffered saline (PBS), 50 µl of hydrogen peroxide, and 10 µl of luminol reagent. The negative control was a mixture of 400 µl of PBS and 10 µl of luminol reagent. Both positive and negative controls were prepared immediately before use. Four hundred µl PBS was used to dilute 20 µl of a semen sample from each aliquot and mixed with 10 µl of luminol reagent. Each sample, including the positive and negative controls, was measured twice, and the crude average value of RLU/sec was corrected by dividing it with the sperm concentration to give the final value of ROS expressed in the unit of RLU/sec/10^6^.

We employed a sperm chromatin dispersion (SCD) test to determine sperm DNA fragmentation using the protocol described by Fernández *et al.* (2003). The principle behind the technique involves sperm embedded in an agarose matrix and lysed to deproteinize the nuclei. Spermatozoa with intact DNA will show extended halos of DNA dispersion. The halos represent relaxed DNA loops, while non-dispersed chromatin displays DNA fragmentation.

### Statistical analysis

Statistical analysis was performed using SPSS version 27. Data are expressed as mean ± standard deviation or median (interquartile range) based on the data distribution. Data were compared by repeated measures analysis of variance (ANOVA) when data distribution was normal or with the Friedman test when normality could not be confirmed. Tukey’s or Dunn’s multiple comparisons test was performed in cases of significant difference. A *p*-value of <0.05 was considered statistically significant.

## RESULTS

Thirty normozoospermic semen samples were included in this study. Table 1 shows the patients’ ages and preprocessing sperm parameters. Post-cryopreservation sperm demonstrated a significant decrease in motility and viability and significantly increased ROS levels in both vitrification and liquid nitrogen vapor freezing. Sperm morphology and DNA fragmentation were comparable in post-cryopreservation sperm and controls (Table 2).

**Table 1:** Baseline characteristics and sperm parameters.

Parameters	Mean±SD
Age (years)	33.0±5.5
Volume (ml)	2.4±1.1
Sperm count (million/ml)	61.7±40.1
Total motility (%)	65.5±14.7
Progressive motility (%)	47.8±13.6
Normal morphology (%)	8.6±4.1

**Table 2:** Sperm motility, viability, morphology, ROS levels, and DNA integrity in control and post-cryopreservation samples, with or without astaxanthin supplementation in cryoprotective media.

Parameters (N=30)	Control	Vitrification		Liquid nitrogen vapor	
		V	V+ATX	L	L+ATX
Total motility (%)	86.0±8.5	69.0±9.5*	77.6±8.9*,†	47.7±14.6*	57.0±13.3*,‡
Progression motility (%)	81.1±10.2	60.2±10.4*	69.5±10.5*,†	39.0±14.5*	48.5±13.6*,§
Viability (%)	80.1±12.5	65.9±11.1*	75.0±11.9†	64.2±9.3*	71.2±12.5*
Morphology (%)	10.5 (6.4-18.2)	9.5 (7.8-12.1)	10.8 (8.8-12.7)	9.5 (8.0-13.1)	10.5 (9.1-12.8)
ROS (RLU/sec/10^6^)	5.8 (4.8-8.8)	10.6 (9.4-16.0) ‖	4.7 (2.6-8.3) ¶	10.3 (7.9-18.6)**	4.6 (3.3-10.5)††
DNA fragmentation (%)	26.5±13.1	27.1±10.4	25.6±12.1	27.2±14.0	25.5±11.4

Repeated measure ANOVA: *p<0.001 *vs.* control, †*p*=0.020 *vs.* V, ‡*p*=0.010 *vs.* L, §*p*=0.020 *vs.* L

Friedman test: l*p*=0.020 *vs.* control, ¶*p*<0.001 *vs.* V, **p=0.030 *vs.* control, ††*p*=0.004 *vs.* L

Abbreviation: V=vitrification, L=liquid nitrogen vapor freezing, ATX=astaxanthin.

### Astaxanthin and Vitrification

A significant increase in sperm total motility (77.6±8.9% *vs*. 69.1±9.5%, *p*=0.020), progressive motility (69.5±10.5% *vs*. 60.2±10.4%, *p*=0.020), and viability (75.0±11.9% *vs*. 65.9±11.1%, *p*=0.020), and a significant decrease in ROS levels (4.7 (2.6-8.3) RLU/sec/10^6^ *vs*. 10.6 (9.4-16.0) RLU/sec/10^6^, *p*<0.001) were observed in the astaxanthin-supplemented group compared to the group without astaxanthin supplementation (Table 2). No difference was seen in sperm morphology (10.8% (8.8-12.7) *vs*. 9.5% (7.8-12.1)) or DNA fragmentation (25.6±12.1% *vs*. 27.1±10.4%) in warmed sperm with or without as-taxanthin supplementation (Table 2). Vitrification did not impair VAP, VSL, VCL, ACH, STR, or LIN. BFC increased significantly in post-vitrified sperm with and without astaxanthin supplementation compared to controls (*p*<0.005, Table 3). The addition of astaxanthin in the cryopreservation media did not affect sperm kinematics.

**Table 3:** Sperm kinematics in control and post-cryopreservation sperm samples in cryoprotective media with or without astaxanthin supplementation.

Parameters (N=30)	Control	Vitrification		Liquid nitrogen vapor	
		V	V + ATX	L	L + ATX
VAP	62.8±10.0	57.5±9.1	60.8±15.7	52.1±8.7*	53.4±8.5*
VSL	49.5±10.0	46.6±7.9	66.5±9.2	43.5±7.3	44.5±8.0
VCL	106.1±20.0	97.7±17.7	97.4±17.4	90.7±14.8*	94.5±14.9
ALH	5.8±1.1	5.4±1.0	5.6±1.3	4.9±0.9*	5.2±1.1
BFC	24.7±3.9	29.1±5.2*	30.0±5.6*	29.1±4.3*	29.0±4.6*
STR	78.7±6.4	80.9±4.3	81.1±5.7	80.8±4.7	79.8±5.6
LIN	49.2±6.7	50.4±5.3	50.3±7.3	47.5±5.5	47.4±5.4

Repeated measure ANOVA: *p<0.005 vs. control

Abbreviation: V = vitrification, L = liquid nitrogen vapor freezing, ATX = astaxanthin.

### Astaxanthin and liquid nitrogen vapor freezing

A significant increase in sperm total motility (57.0±13.3% *vs*. 47.7±14.6%, *p*=0.010), progressive motility (48.5±13.6% *vs*. 39.0±14.5%, *p*=0.020), and a significant decrease in ROS levels (4.6 (3.3-10.5) RLU/ sec/10^6^ *vs*. 10.3 (7.9-18.6) RLU/sec/10^6^, *p*=0.004) were observed in the astaxanthin-supplemented group compared to the group without astaxanthin supplementation (Table 2). There were no differences in sperm viability (71.2±12.5% *vs*. 64.2±9.3%), morphology (10.5% (9.1-12.8) *vs*. 9.5% (8.0-13.1)) or DNA fragmentation (25.5±11.4% *vs*. 27.2±14.0%) in thawed sperm with or without astaxanthin supplementation (Table 2). The liquid nitrogen vapor method did not affect VSL, STR, or LIN, while VAP, VCL, and ALH decreased significantly, and BFC increased significantly after freezing (*p*<0.005) (Table 3). Astaxanthin supplementation did not affect sperm kinematics.

## DISCUSSION

Cryopreservation precipitates many deleterious consequences on most sperm parameters, including sperm motility, viability, ROS level, and DNA fragmentation due to damage to the plasma membrane and intracellular organelles and mitochondria by lipid peroxidation (Guthrie & Welch, 2012; Ozimic *et al.*, 2023). In this study, we proposed that adding the antioxidant astaxanthin, which has been used extensively in many fields, might improve the cryopreservation process of human sperm in parameters such as increased post-thaw motility, overall survival, and decreased ROS production. Our PubMed search used the terms astaxanthin and human sperm cryopreservation and found only two publications on the subject. The two used liquid nitrogen vapor freezing as a cryopreservation technique. They showed that astaxanthin significantly improved post-thaw sperm motility (Dede & Saylan, 2022; Ghantabpour *et al.*, 2022) and viability (Ghantabpour *et al.*, 2022), and decreased ROS level (Ghantabpour *et al.*, 2022) and sperm DNA fragmentation (Dede & Saylan, 2022). There is no study using the vitrification technique. Therefore, we focused on the effect of astaxanthin supplementation on cryoprotective media in the two sperm cryopreservation techniques, liquid nitrogen vapor freezing, and vitrification.

Similar to previous studies in humans (Dede & Saylan, 2022; Ghantabpour *et al.*, 2022), pigs (Lee & Kim, 2018), and boar semen (Basioura *et al.*, 2018), we confirmed the beneficial effects of astaxanthin in post-cryopreservation sperm motility. Astaxanthin improved sperm viability in both groups, but this increase was significant only in the vitrification group. Our study supported previous research (Lee & Kim, 2018; Len *et al*., 2019; Evangelista-Vargas & Santiani, 2017; Santiani *et al*., 2014) in that cryopreservation increased ROS levels. We found increases in ROS levels in both cryopreservation techniques. In agreement with previous studies in pigs (Lee & Kim, 2018), astaxanthin significantly reduced ROS during cryopreservation.

ROS levels decreased to baseline in both supplemented groups, indicating astaxanthin’s potent antioxidant properties. Our review of the literature showed that astaxanthin spans itself across the cell membrane by using its polar end groups overlapping the polar boundary zones of the membrane. As such, it conducts electrons out of the membrane to other antioxidants, especially human serum albumin, outside the membrane without being destroyed (Kidd, 2011). This might explain the tremendous antioxidant capacity of astaxanthin observed in this study.

In our study, freezing and thawing did not affect normal sperm morphology, unlike what O’Connell *et al*. (2002) found. This could be explained by the fact that they cryopreserved neat semen samples, while we cryopreserved sperm after density-gradient preparation. Another study showed that cryopreservation of processed sperm samples resulted in better sperm quality and fewer apoptotic sperm cells than cryopreservation of neat sperm (Petyim *et al.*, 2014).

Consistent with our previous studies (Vutyavanich *et al.*, 2010; 2012), we found no significant increase in sperm DNA fragmentation after cryopreservation by either cryopreservation method. However, this is still controversial, as some authors (Donnelly *et al.*, 2001; de Paula *et al.*, 2006; Cankut *et al.*, 2019; Le *et al.*, 2019) reported substantial alterations in sperm DNA integrity after cryopreservation. In contrast, others have indicated that cryopreservation does not affect the stability of sperm DNA (Duru *et al.*, 2001; Schuffner *et al.*, 2001; Isachenko *et al.*, 2004; Paasch *et al.*, 2004; Rayea *et al.*, 2020). Opposing views can arise from many uncontrolled factors, such as subject variability, cryopreservation technique, and the method used in sperm DNA fragmentation evaluation. Studies that reported significant increases in DNA fragmentation often employed cryopreservation of neat sperm and had poor sperm motility after cryopreservation. One caveat was that dead sperm might influence the results of the assays. In future studies, we should evaluate DNA fragmentation only on motile or morphologically normal sperm cells instead of the whole sperm preparation (Liu & Liu, 2013; Palermo *et al.*, 2014; Vandekerckhove, 2017).

Theoretically, we could have performed a similar study in vivo by prescribing oral astaxanthin. However, the substance is poorly absorbed orally, and it would take months to achieve protective effects in semen. Individual variation in absorption of the compound must also be considered. In addition, there are no data on the bioavailability of astaxanthin in semen. We, therefore, chose to perform an in vitro study to bypass these issues. Interestingly, a randomized controlled trial showed that oral intake of astaxanthin for three months positively affected sperm parameters and fertility (Comhaire *et al.*, 2005). Unfortunately, they did not study sperm parameters post-cryopreservation in supplemented and non-supplemented subjects.

Only normospermic males were included in this study because they provide adequate sperm cells for reliable assessment of sperm parameters, DNA fragmentation, and ROS. The actual target population is likely to be infertile males with abnormal semen parameters, in whom a 10-20% relative improvement in sperm parameters post-cryopreservation might be clinically relevant. The reliability problem in outcome assessment in this group of patients could be partially overcome by recruiting more subjects and using more sophisticated techniques to assess outcome parameters. For example, live fluorescent dye could be used during CASA evaluation of count and motility to differentiate them from debris. An imaging flow cytometry could be used for DNA fragmentation assessment in live sperm by double fluorescent staining with TdT-mediated fluorescein-dUTP nick end labeling for TUNEL and Far-Red fluorescent stain (L10120) to differentiate live/dead sperm simultaneously.

In this preliminary study, we did not evaluate sperm function tests or have data on live births of men taking astaxanthin supplementation. Nevertheless, the consistent results from our in-vitro study and the in-vivo study by Comhaire *et al.* (2005) strongly suggest a beneficial effect for astaxanthin. Further research on astaxanthin supplementation should be performed, especially in men with a low sperm count.

Our study was affected by several limitations. The number of participants included in the study is considered too low to detect a more comprehensive array of differences in the analyzed parameters. Therefore, the number of participants should be increased to detect the differences in DNA fragmentation. Secondly, the samples were taken only from individuals with normal semen who met the WHO criteria for normozoospermia. Individuals who might potentially benefit from astaxanthin – men with subpar sperm parameters – were not included in the study. Therefore, we failed to express the general usefulness of astaxanthin. Thirdly, sperm function tests were not performed, and supplemented samples were not used to achieve pregnancy, a potential benefit missed by the study. Fourthly, the dosage was derived from our pilot study in which a difference in post-thaw motility was seen. Dosage variation might be valid in different methods of cryopreservation and other populations. The effect of personalized doses of the antioxidative agent may be analyzed in future studies.

In conclusion, adding astaxanthin to the cryoprotection media had a beneficial effect and improved post-cryopreservation sperm motility, decreased ROS levels in both vitrification and liquid nitrogen vapor freezing, and improved sperm viability in the vitrification technique.
